# Effect of Molybdenum (Mo) Addition on Phase Composition, Microstructure, and Mechanical Properties of Pre-Alloyed Ti6Al4V Using Spark Plasma Sintering Technique

**DOI:** 10.3390/molecules26102894

**Published:** 2021-05-13

**Authors:** Murugesan Rajadurai, Ayyapparaj Muthuchamy, A. Raja Annamalai, Dinesh K. Agrawal, Chun-Ping Jen

**Affiliations:** 1 Department of Aeronautical Engineering, Mahendra Engineering College (Autonomous), Namakkal 637503, Tamil Nadu, India; mrajadurai700@gmail.com; 2 Department of Metallurgical and Materials Engineering, National Institute of Technology Tiruchirappalli, Tiruchirappalli 620015, Tamil Nadu, India; muthuchamy@nitt.edu; 3 Centre for Innovative Manufacturing Research, Vellore Institute of Technology, Vellore 632014, Tamil Nadu, India; raja.annamalai@vit.ac.in; 4 Materials Research Institute, The Pennsylvania State University, University Park, PA 16802, USA; dxa4@psu.edu; 5 Department of Mechanical Engineering and Advanced Institute of Manufacturing for High-Tech Innovations, National Chung Cheng University, Chia-Yi 62102, Taiwan

**Keywords:** Ti6Al4V-xMo, microstructure, mechanical properties, yield strength, ultimate tensile strength, ductility, spark plasma sintering

## Abstract

The effect of molybdenum additions on the phases, microstructures, and mechanical properties of pre-alloyed Ti6Al4V was studied through the spark plasma sintering technique. Ti6Al4V-xMo (where x = 0, 2, 4, 6 wt.% of Mo) alloys were developed, and the sintered compacts were characterized in terms of their phase composition, microstructure, and mechanical properties. The results show that the equiaxed primary alpha and Widmänstatten (alpha + beta) microstructure in pre-alloyed Ti6Al4V is transformed into a duplex and globular model with the increasing content of Mo from 0 to 6%. The changing pattern of the microstructure of the sample strongly influences the properties of the material. The solid solution hardening element such as Mo enhances mechanical properties such as yield strength, ultimate tensile strength, ductility, and hardness compared with the pre-alloyed Ti6Al4V alloy.

## 1. Introduction

Titanium and its alloys exhibit excellent mechanical properties such as high strength and stiffness, high plasticity, and light weight, with outstanding corrosion resistance [1,2,3,4,5]. Hence, they (especially Ti6Al4V) are finding increased applications in automobile engines, biomedical devices, airplanes, rockets and missile components, and marine fields [6,7,8,9,10]. However, the cost of material development and machining of Ti and its alloy components is too high for many of these engineering applications [11,12,13,14,15]. Pure commercial Titanium does not meet its increased demand. Therefore, Ti alloys are modified to have improved mechanical properties by adding different stabilizers such as neutral (Tin and Zirconium) stabilizers, alpha (Aluminum, Oxygen, Nitrogen, and Carbon) stabilizers, beta isomorphous (Molybdenum, Vanadium, Tantalum, Niobium) stabilizers, and beta eutectoid (Iron, Manganese, Chromium, Cobalt, Nickel, Copper, Silicon, and Hydrogen) stabilizers [16,17,18]. The alpha stabilizers exhibit good corrosion resistance; beta isomorphous elements exhibit complete solubility with the Ti and form an intergranular beta phase that limits the ductility; beta eutectoid elements exhibit incomplete solubility with Ti and make it brittle [19,20,21]. According to the hardening effects of the isomorphous beta stabilizers (Mo, V, Ta, Nb), Mo has been proved to be more effective in strengthening Ti alloys than V, Ta, and Nb addition [22,23,24]. Most researches involve the study of phase and microstructural characteristics, mechanical properties, the formation of martensite alpha, and the effect of the elemental composition of Mo on the deformation behavior of different proportions of Ti-Mo alloy and found that the phase fractions, microstructures, and mechanical properties are different for different composition of Mo in Ti-Mo alloys [25,26,27,28,29,30,31,32]. It has been proved that the addition of Mo to the Ti alloy enhances its mechanical properties very effectively, but when increasing the Mo content to 10 wt.% and above, the phases become entirely converted into the beta phase [33,34,35]. Although the connection between phase fractions and mechanical properties in Ti-Mo has not been entirely comprehended, until now, researchers are becoming involved in the investigation between pure Titanium with molybdenum. The traditional titanium alloy production methods were casting and wrought [36,37], and additive manufacturing methods have significant advantages such as being free from defects, fine microstructure, better diffusion, and cost reduction [38]. Some of the special sintering techniques used to process the Grade V Ti alloys are hot isostatic pressing (HIP), spark plasma sintering (SPS), and microwave sintering [39,40,41,42,43,44]. Spark plasma sintering techniques have various advantages over other sintering techniques, such as a fast heating rate and less time of sintering, and the sample effectively achieved a fine microstructure [45,46,47,48]. This research work deals with a series of Ti6Al4V-xMo (where x = 0, 2, 4, 6 wt.% of Mo) alloys that have been characterized by using SPS techniques. The main research objective is to investigate the influence of molybdenum additions on the phases, microstructures, and mechanical properties of pre-alloyed Ti6Al4V powder material.

## 2. Results and Discussion

### 2.1. Densification

The powder particle size and the sintering parameters such as temperature, pressure, and holding time influence the sample’s densification [49]. The densification results of the sample Ti6Al4V-xMo (where x = 0, 2, 4, 6 wt.% of Mo) alloys are listed in Table 1. In this study, the pre-alloyed Ti6Al4V combination accomplished the most excellent sintered density of 4.417 and 97.9% of the relative density. Adding molybdenum content from 2% to 6% in the pre-alloyed Ti6Al4V diminishes the relative density from 96.8 to 95.3% (Table 1). The reason is that the molybdenum has a higher density (10.28 g/cm^3^) than the pre-alloyed Ti6Al4V (4.42 g/cm^3^) powder material in the mixtures Ti6Al4V-xMo (where x = 0, 2, 4, 6 wt.% of Mo) and functions as a strong solid solutions strengthening agent, limiting the dispersion between the particles. Incomplete diffusion develops the pores in the sample, consequently reducing the sintered density.

### 2.2. Phase Composition and Microstructural Development

**Figure 1:**
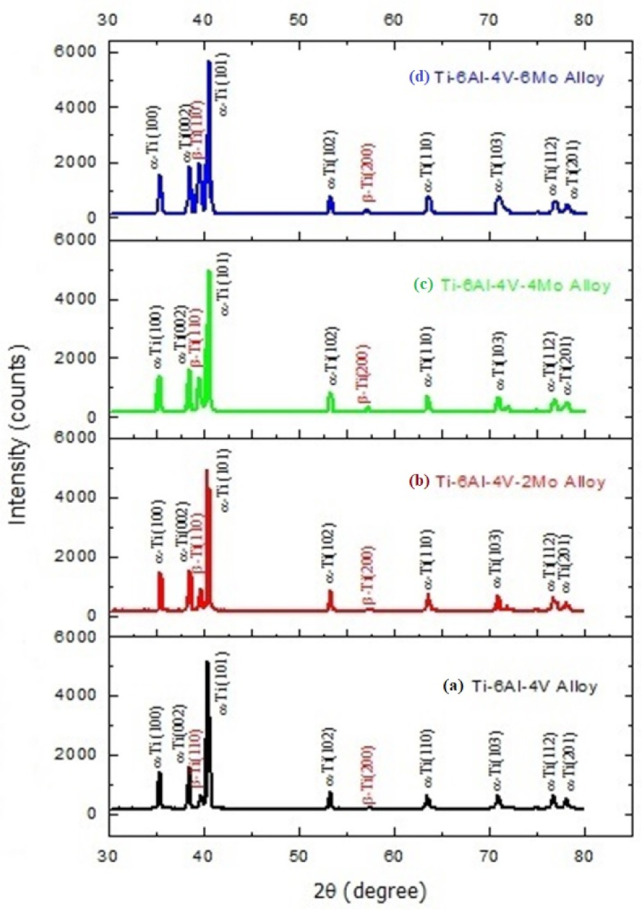
XRD pattern results of the Titanium alloys: (**a**) pre-alloyed Ti6Al4V, (**b**) Ti6Al4V-2Mo, (**c**) Ti6Al4V-4Mo, and (**d**) Ti6Al4V-6Mo.

**Figure 2:**
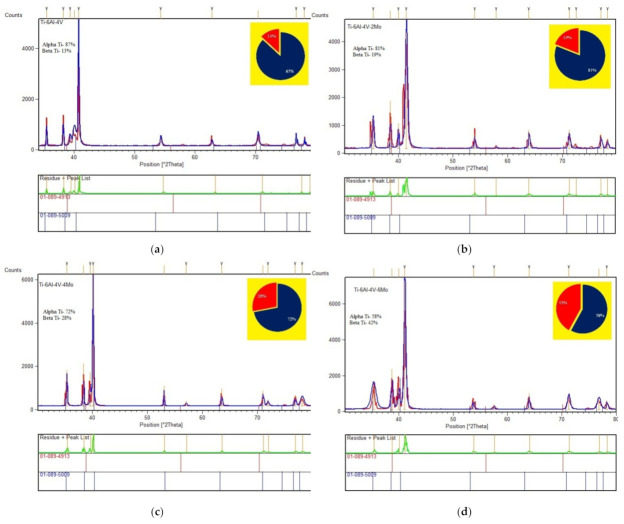
XRD patterns with phase fractions results of Titanium alloy samples: (**a**) pre-alloyed Ti6Al4V alloy, (**b**) Ti6Al4V-2Mo alloy, (**c**) Ti6Al4V-4Mo alloy, and (**d**) Ti6Al4V-6Mo alloy.

The XRD patterns of the Titanium alloys Ti6Al4V-xMo (where x = 0, 2, 4, 6 wt.% of Mo) are presented in Figure 1. X-Pert High Score Plus software was used to determine the titanium alloys’ phase fraction (Ti6Al4V-xMo). With the help of the JCPDS file (No. #010895009—α Ti; No. #010894913—β Ti) in the software X-Pert High Score Plus, the peaks from the patterns are fixed and coordinated, then the related phase fraction images of Titanium alloys Ti6Al4V-xMo (where x = 0, 2, 4, 6 wt.% of Mo) appear in Figure 2. The Ti6Al4V-xMo alloy was indexed from this qualitative measurement, and the related β phases were appropriately reflected from reflections 110 and 200, respectively. From these XRD pattern results, there is no evidence of moving of α peaks. However, the β phase movements of peaks occur when including the β stabilizer Mo from 2 to 6 wt.%, demonstrating that proof of lattice distortion happens [50,51]. One can see that the β Phase is available in the relating lattice deformation due to increasing the addition of β stabilizer Mo. The lattice factors of Ti6Al4V alloy are generally determined from Figure 1, and the indexed (110 and 200) beta phase reflections is about 0.3228 nm, which is similar to that specified to β-Ti, for example, 3.283 A (JCPDS file no. #010894913). Likewise, the lattice factors are increased with Mo’s addition from 2 to 6 wt.% in pre-alloyed Ti6Al4V powder material (Ti6Al4V-2Mo, Ti6Al4V-4Mo, and Ti6Al4V-6Mo). The listed values are 0.3232 nm, 0.3243 nm, and 0.3251 nm. This indicates an increase of values of 0.71%. This increasing trend of lattice parameters and the β phase fraction characterizes the expansion of β stabilizer Mo content from 2 to 6 wt.%, acting as the solid-solid solution strengthening agent in the pre-alloyed Ti6Al4V alloy. The results (Figure 2a–d, phase fraction estimations) of the individual phase part esteem (α, β) were recorded in Table 2. This represents that the number of α and β fractions are varied concerning the amount of molybdenum (Mo) addition in pre-alloyed Ti6Al4V powder material. The β stabilizer Mo is influenced to increase the amount of β phase from 13 to 42% in the alloy Ti6Al4V-xMo (where x = 0, 2, 4, 6 wt.% of Mo).

**Figure 3:**
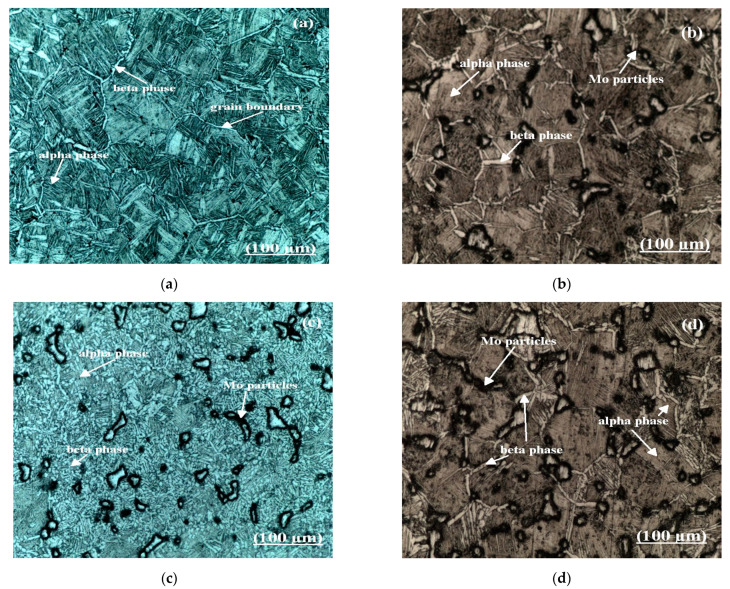
Optical microstructures of the Titanium alloy: (**a**) pre-alloyed Ti6Al4V, (**b**) Ti6Al4V-2Mo, (**c**) Ti6Al4V-4Mo, and (**d**) Ti6Al4V-6Mo.

The microstructures of Ti6Al4V-xMo (where x = 0, 2, 4, 6 wt.% of Mo) are shown in Figure 3. The microstructure of the pre-alloyed Ti6Al4V comprises of equiaxed primary α, and the α lamellae colonies are developed within the β phase grains consisting of the so-called “Widmanstätten” or “Basketweave” microstructure distributed all over the specimen. This microstructure was developed from the single-phase β phase field with the effect of cooling of the sample. When 2% of molybdenum is included in the pre-alloyed Ti6Al4V alloy, a duplex microstructure was created in the specimen entirely. Addition of molybdenum will, in general, lead to the improvement of β phase, increasing the thickness of the β phase grain boundary and the gathering of α colonies created in the β phase locale. The high-density β stabilizer molybdenum’s contents are randomly distributed on the specimen and diffused with the alloying elements from the β phase separately [51]. The dark color in the microstructure consists of β stabilizer molybdenum (Mo) content surrounding the β phases due to diffusion.

Similarly, when Mo content is increased from 2 to 4% in the pre-alloyed Ti6Al4V, the large size of β grains consisting of duplex microstructure are converted into the globular microstructure. Increasing the β stabilizer Mo reduces the transition temperature (α + β → β) in the pre-alloyed Ti6Al4V and tends to a large number of small size β phase grains that are developed in the specimen, and the spheroid particle of Mo substance is randomly dispersed in the specimen [50]. At last, for the 6% additions of Mo in pre-alloyed Ti6Al4V alloy, a mixed combination of duplex and globular microstructure was developed, consisting of a large amount of β stabilizer Mo content that tends to increase the β grain boundary, and the equiaxed α phases were developed within the β region. From these microstructure images, the β stabilizer Mo substance will, in general, leads to the change of microstructure from “Widmanstätten” or “Basketweave” into a mixed combination of duplex and globular microstructures in pre-alloyed Ti6Al4V material.

**Figure 4:**
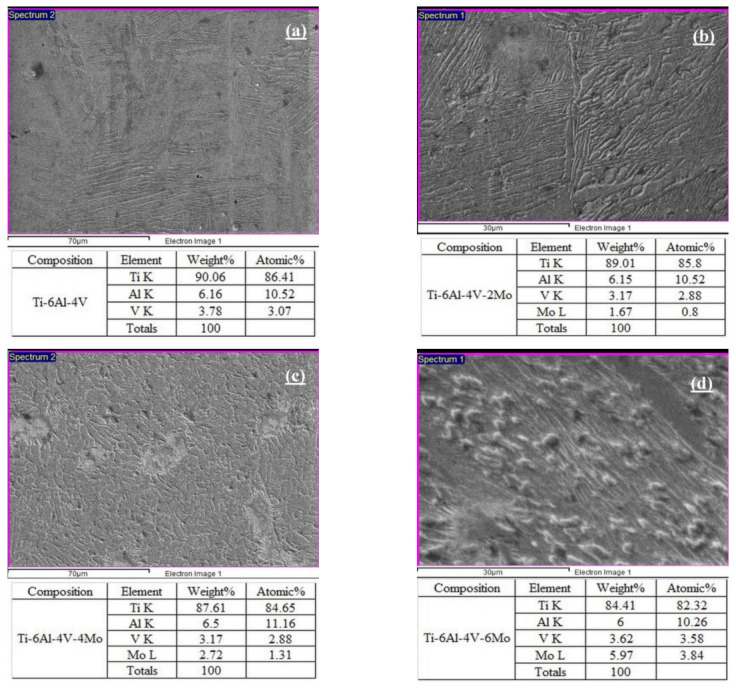
SEM images and EDAX analysis of the Titanium alloys: (**a**) pre-alloyed Ti6Al4V, (**b**) Ti6Al4V-2Mo, (**c**) Ti6Al4V-4Mo, and (**d**) Ti6Al4V-6Mo.

The SEM and EDAX analysis of Ti6Al4V-xMo affirms the phases, microstructures with α and β phase grains, grain boundary, and elemental composition accurately. Figure 4 exhibits the SEM images with EDAX analysis of elemental compositions of Ti6Al4V-xMo, which reveal incomplete densification, variation of porosity levels, and α and β particles with grain boundary developments precisely. The β stabilizer molybdenum additions in pre-alloyed Ti6Al4V greatly vary the phase developments with pores shown in Figure 4b. Without the addition of molybdenum in pre-alloyed Ti6Al4V, the SEM image resembles clear α and ß phase development. However, the β stabilizer molybdenum contents are added from 2 to 6 wt.% in the pre-alloyed Ti6Al4V, the microstructure transforms with the diffusion of molybdenum (Mo) and the variation of pores. From these SEM results, the β stabilizer molybdenum’s contents are strongly influenced by the microstructural changes in pre-alloyed Ti6Al4V. EDAX results show that the presence of alloying elements Titanium (Ti), Aluminum (Al), Vanadium (V), and Molybdenum (Mo) in the specimen Ti6Al4V-xMo through elemental mapping with their wt.% and atomic % are determined precisely, and corresponding elemental values are mentioned in Figure 4. From this, elemental mapping results from the addition of β stabilizer molybdenum from 2 to 6 wt.% in pre-alloyed Ti6Al4V showed an increased number of β phases, which is confirmed by the increase of wt.% and atomic % values.

### 2.3. Mechanical Properties

**Figure 5:**
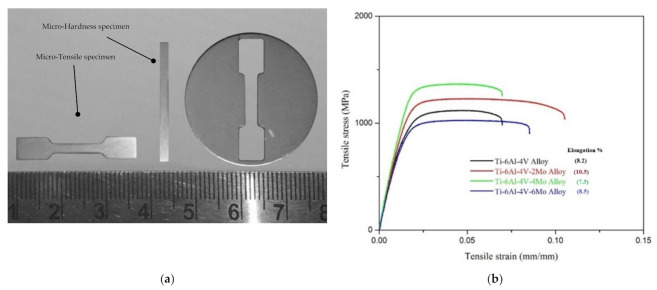
(**a**) Micro-tensile and micro-hardness specimen; (**b**) micro-tensile testing results.

The micro-tensile testing results of the sample Ti6Al4V-xMo alloys appear in Figure 5. The mechanical properties such as YS, UTS, and elongation % (ductility) are noted in Table 2. From the mechanical properties data, the specimen without the addition of molybdenum in pre-alloyed Ti6Al4V shows lower values (993MPa of YS, 1120MPa of UTS, and 8.2% of elongation %) than with the addition of molybdenum. The sample with the addition of 2% molybdenum in pre-alloyed Ti6Al4V shows improved mechanical properties over pre-alloyed Ti6Al4V (UTS from 1120MPa to 1230MPa, ductility from 8.2% to 10.5%) [51]. Similarly, the sample with a 4% addition of molybdenum (Mo) in pre-alloyed Ti6Al4V shows the highest mechanical values, from 1230 MPa to 1368 MPa UTS, compared with all other samples, but the ductility is decreased to 7.3%. Finally, the sample with a 6% addition of molybdenum (Mo) shows decreased mechanical values (UTS 1038MPa) compared with all other samples. From these results, the addition of β stabilizer molybdenum (Mo) in pre-alloyed Ti6Al4V enormously modifies the alloys’ microstructures and mechanical properties. Referenced above, the sample Ti6Al4V with 2% Mo additions changed the microstructure from Widmanstätten into the duplex (bimodal) model microstructure. In general, the microstructural variations significantly impact the mechanical properties of the material. The samples’ lamellar microstructures show lower mechanical properties than the bimodal microstructure; the same parameters are reflected from the sample Ti6Al4V-2Mo (bimodal microstructure) because of the β stabilizer molybdenum; therefore, the sample shows higher UTS and ductility than the pre-alloyed Ti6Al4V. Likewise, in Ti6Al4V-4Mo, the microstructure is changed into a mixed combination of the globular and duplex model with various pores, so it has high yield strength and UTS, yet the ductility is diminished because of the pores. Finally, the alloy Ti6Al4V-6Mo shows lower yield strength and UTS than all other alloys because the large amount of Mo solutes constrains the dispersion between particles and forms pores with restricted mechanical values.

The microhardness testing results of the alloys Ti6Al4V, Ti6Al4V-2Mo, Ti6Al4V-4Mo, and Ti6Al4V-6Mo are recorded in Table 2. From the entire sample of Ti6Al4V-xMo, Ti6Al4V-6Mo alloy shows the highest hardness (370 HV_500_) value. The hardness values increased from 330 HV_500_ to 370 HV_500_ with the increasing addition of β stabilizer molybdenum (Mo) content from 2 to 6 wt.% in pre-alloyed Ti6Al4V. Results show that Mo as a solid-solid solution hardening agent in pre-alloyed Ti6Al4V increasing with the addition of Mo content in pre-alloyed Ti6Al4V significantly changes the microstructural effects from a large number of α phase fractions into β phase fractions. Due to these increased β phase fraction values, the specimen acted as high hardness.

**Figure 6:**
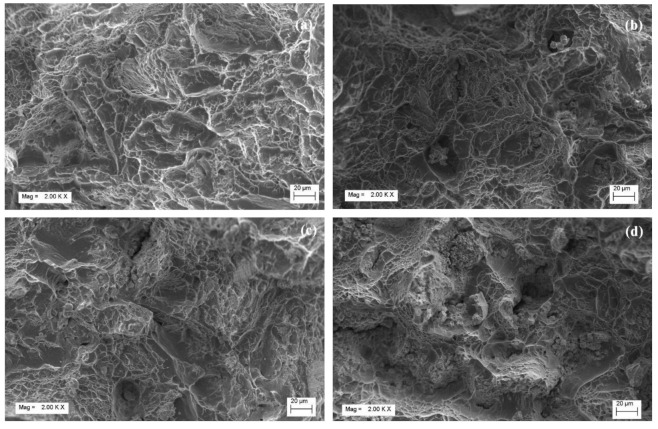
Fractography images of the Titanium alloys: (**a**) pre-alloyed Ti6Al4V, (**b**) Ti6Al4V-2Mo, (**c**) Ti6Al4V-4Mo, and (**d**) Ti6Al4V-6Mo.

The fractography images of the Titanium alloy specimens Ti6Al4V, Ti6Al4V-2Mo, Ti6Al4V-4Mo, and Ti6Al4V-6Mo are displayed in Figure 6. In Figure 6a, without the addition of β stabilizer molybdenum in pre-alloyed Ti6Al4V, the dimples are appropriately distributed on the fractured zone and show that a large amount of plastic deformation takes place in the alloy Ti6Al4V, which is evidence of the ductile mode of fracture taking place in the specimen. Similarly, in Figure 6b, the small amount of Ti6Al4V-2Mo alloy shows that Mo refined the dimple size and shape; consequently, the specimen has high plasticity with a small number of pores. However, the specimens Ti6Al4V-4Mo and Ti6Al4V-6Mo alloy in Figure 6c,d show incomplete densification because of the high-density material molybdenum, and the pores are apparent. The incomplete densification and many pores are mainly because of insufficient activation energy between the particles’ contact points, so the material behaves like a brittle mode of failure with less elongation.

## 3. Materials and Methods

### 3.1. Powder Morphology

**Figure 7:**
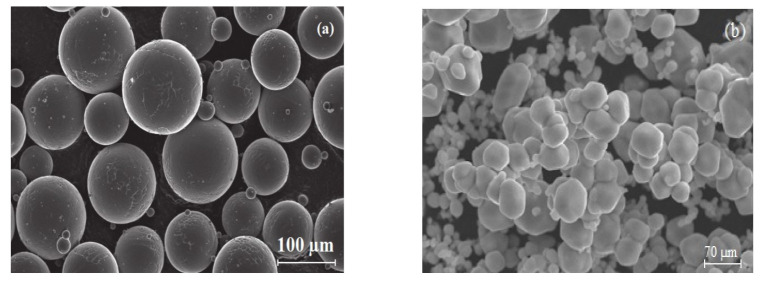
Scanning electron microscope images of powder material: (**a**) pre-alloyed Ti6Al4V and (**b**) molybdenum (Mo).

The starting powders utilized in this work are pre-alloyed Ti6Al4V from AP&C, Canada, and Molybdenum from Sigma-Aldrich, Bangalore, India. The powder morphologies were investigated by scanning electron microscopy (SEM), which shows that the spherical powder particles of pre-alloyed Ti6Al4V powders are uniformly distributed (particle size 45–106 µm), the majority of the molybdenum powder particle sizes are less than 74 µm (95%), and a small number of particle sizes are in the range of 74–149 µm (5%) (Figure 7). The chemical compositions of as-received powder materials are listed in Table 3.

### 3.2. Alloy Preparations

Powder blends of Ti6Al4V-xMo (where x = 0, 2, 4, 6 wt.% of Mo and balances pre-alloyed Ti6Al4V) were alloyed mechanically through high-energy ball milling for 1 h. The nominal compositions of the wt.% of Ti6Al4V and Mo are mentioned (Table 4).

### 3.3. Sintering

The mixed powders of Titanium alloy samples were packed with die assembly and placed inside the spark plasma sintering unit (Dr. Sinter, model no. SPS-625, SPS syntex incorporation, Kawasaki, Japan). High-density graphite die and punches were used. The cylindrical die consists of an inside diameter of 20 mm, outside diameter of 50 mm, and height of 50 mm. A 0.15 mm-thick graphite foil was inserted between the graphite die’s inner surface and the powder material, and on the top and bottom faces of the graphite plungers, to facilitate easy removal of the sintered sample. Graphite felt was used to cover the die to minimize the heat losses by thermal radiation at high temperatures. The samples were sintered at a temperature of 900 °C with a dwell time of 2 min under 50 MPa pressure load, heating rate 100 °C/min, and vacuum 1.34 × 10^−1^ Pa. The optical pyrometer was used to monitor the sintering temperatures. Finally, the samples (disk-shaped) were fabricated from 30 g of mixed compositions with 30 mm diameter and 8 mm in height. The cylindrical shapes of sintered compacts were machined using electrodischarge machining to characterize and perform mechanical testing.

### 3.4. Characterization

After machining, all the samples were correctly fixed in a grinding and polishing machine with appropriate grit papers (120, 220, 400, 600, and 1000) and diamond paste (3–4 μm and 0.5–1 μm). The ASTM E407 method was followed to complete the etching process with the etchant Kroll’s solutions consisting of 3 ml HF, 6 mL HNO_3_, and 100 mL H_2_O. The scanning electron microscope equipped with energy dispersive spectrometry (Zeiss Evo 50, Carl Zeiss SMT Ltd., Cambridge, UK) was used to characterize the microstructures and the chemical composition of the sintered samples. The SEM images and chemical proportions of the samples are obtained from the polished sample. An optical microscope (LEICA DM2500, Wetzlar, Germany) to obtain the digital image was used to obtain the specimen’s microstructure. The phase composition of the samples was determined using an XRD spectrometer (Bruker D8 Discover XRD machine) with the corresponding anode material, radiation of the wavelength (Å), step size (°2θ), and scan rate(s) Cu, 1.54060 (K-α1), 1.54443 (K-α2), 1.39225 (K-β), 0.0130 and 48.1950, respectively. The phase fraction analysis was done by the software X-Pert High Score Plus through the Rietveld analysis methods. Acetone was used to clean the samples’ fractured area and then viewed under a scanning electron microscope for fractography analysis.

### 3.5. Testing

The sintered specimen’s density was obtained by the Archimedes method. The mechanical properties such as yield strength, ultimate tensile strength, and the microtensile sample’s ductility were measured using the INSTRON universal testing machine with an initial strain rate of 0.2 mm s^−1^ (2 mm/minimum crosshead speed) of full-load 10 kN. To ensure reproducibility, the mechanical testing of five samples was conducted at room temperature. The sintered compacts microhardness was carried out from the instrument ECONOMET-VH-1MD with the diamond indenter angle set to 136°. The testing was done on a polished surface area, and the results consist of 49 indentations (mean, standard deviation) of 7 × 7 matrices with 10 μm of space between the indents. By using the scanning electron microscope, the fractured area of the specimen was examined.

## 4. Conclusions

Ti6Al4V-xMo (where x = 0, 2, 4, 6 wt.% of Mo) alloys were prepared using the spark plasma sintering method, and uniform elemental distribution microstructures were obtained. From this work, the following conclusions can be made.

- Molybdenum addition of 2 to 6 wt.% in pre-alloyed Ti6Al4V changes the microstructure from equiaxed primary alpha and Widmanstätten (alpha + beta) into a duplex and Globular model with a variation of pores.
- Mo acts as an effective solid solution strengthening agent, which is reflected in the improved mechanical properties of the alloys.
- Ti6Al4V-4Mo exhibits good mechanical values such as high yield stress (1174 MPa) and UTS (1368 MPa) with a lower percentage of elongation (7.3) than the remaining three alloys Ti6Al4V, Ti6Al4V-2Mo, Ti6Al4V-6Mo; the microhardness of Ti6Al4V-6Mo has the highest value (370 HV_500_) among all compositions.
- From the fractography images, the high-density molybdenum (Mo) content forms the pore, modifies the shape and size of dimples, and indicates the brittle mode of fracture with a lower elongation %.

## Figures and Tables

**Table 1:** Relative densities of sintered specimens.

S. No.	Alloy	Theoretical Density (g/cm^3^)	Sintered Density (g/cm^3^)	Relative Density (%)
**1**	Ti6Al4V	4.51	4.41	97.9
**2**	Ti6Al4V-2Mo	4.56	4.41	96.8
**3**	Ti6Al4V-4Mo	4.62	4.45	96.3
**4**	Ti6Al4V-6Mo	4.67	4.45	95.3

**Table 2:** Phase fractions, micro-tensile testing results, and hardness values of Titanium alloy samples.

S. No.	Alloy	% of α Phase	% of β Phase	Yield Stress (MPa)	UTS(MPa)	Elongation %	Avg. Hardness Value (HV_500_)
							
1	Ti6Al4V	87	13	993	1120	8.2	330
2	Ti6Al4V-2Mo	81	19	1073	1230	10.5	340
3	Ti6Al4V-4Mo	72	28	1174	1368	7.3	345
4	Ti6Al4V-6Mo	58	42	885	1028	8.5	370

**Table 3:** Purity, chemical composition, particle size, and source of the raw materials used.

S. No.	Powder Material	Supplier	Purity and Chemical Composition	Powder Particle Size
1	Pre-alloyed Ti6Al4V	AP&C, Canada	Purity > 99.6%; ~88.649% Ti, Al (6.40%), V (4.20%), C (0.01%), O (0.13%), N (0.01%), H (0.001%), Fe (0.21%), and Others (0.39%).	>106 μm (3.8%) ≤ 106 μm > 45 μm (94.6%) ≤45 (1.6%)
2	Molybdenum (Mo)	Sigma-Aldrich, Bangalore, India	Purity 99.95%; ~99.84% Mo, O (0.1%), N (0.01%), C (0.005%), W (0.025%), Fe (0.005%), Ni (0.005%), Cr (0.005%), and Si (0.005%)	≤ 149 μm > 74 μm (5%) ≤74 μm (95%)

**Table 4:** Nominal composition of Titanium alloy samples.

Sr.	Specimen	Nominal Composition	
No.		Ti6Al4V (wt.%)	Mo (wt.%)
1	Ti6Al4V-0Mo	100	0
2	Ti6Al4V-2Mo	98	2
3	Ti6Al4V-4Mo	96	4
4	Ti6Al4V-6Mo	94	6

## Data Availability

Data available on request due to restrictions. The data presented in this study are available on request from the corresponding author. The data are not publicly available due to the on-going work.

## Sample Availability

Sample of the compounds are available from the authors.

## References

[1] Boyer R.R., Briggs R.D. (2005). The Use of β Titanium Alloys in the Aerospace Industry. J. Mater. Eng. Perform..

[2] Yang F., Gabbitas B. (2017). Feasibility of producing Ti-6Al-4V alloy for engineering application by powder compact extrusion of blended elemental powder mixtures. J. Alloys Compd..

[3] Carman A., Zhang L.C., Ivasishin O.M., Savvakin D.G., Matviyc huk M.V., Pereloma E.V. (2011). Role of alloying elements in microstructure evolution and alloying elements behaviour during sintering of a near-β titanium alloy. Mater. Sci. Eng. A.

[4] Esen Z., Bor S. (2011). Characterization of Ti–6Al–4V alloy foams synthesized by space holder technique. Mater. Sci. Eng. A.

[5] Bao J., Yang S., Yang T. (2020). Microstructural evolution, tensile property and dynamic compressive property of FSWed Ti–6Al–4V alloy. Rare Met..

[6] Jabur A.S., Al-Haidary J.T., Al-Hasani E.S. (2013). Characterization of Ni–Ti shape memory alloys prepared by powder metallurgy. J. Alloys Compd..

[7] Chen W., Yamamoto Y., Peter W.H., Clark M.B., Nunn S.D., Kiggans J.O., Muth T.R., Blue C.A., Williams J.C., Akhtar K. (2012). The investigation of die-pressing and sintering behavior of ITP CP-Ti and Ti-6Al-4V powders. J. Alloys Compd..

[8] Brailovski V., Prokoshkin S., Gauthier M., Inaekyan K., Dubinskiy S. (2013). Mechanical properties of porous metastable beta Ti–Nb–Zr alloys for biomedical applications. J. Alloys Compd..

[9] Hagiwara M., Emura S. (2003). Blended elemental P/M synthesis and property evaluation of Ti-1100 alloy. Mater. Sci. Eng. A.

[10] Wang M., Li H.Q., Guo H., Feng L., Liu S.Y., Fang X.Y. (2020). Evolution of microstructure and intervariant boundaries of α phase in electron beam melted and heat-treated Ti–6Al–4V alloy. Rare Met..

[11] Henriques V.A.R., de Campos P.P., Cairo C.A.A., Bressiani J.C. (2005). Production of titanium alloys for advanced aerospace systems by powder metallurgy. Mater. Res..

[12] Chen B.-Y., Hwang K.-S., Ng K.-L. (2011). Effect of cooling process on the α phase formation and mechanical properties of sintered Ti–Fe alloys. Mater. Sci. Eng. A.

[13] Nouri A., Hodgson P.D., Wen C.E. (2010). Effect of process control agent on the porous structure and mechanical properties of a biomedical Ti–Sn–Nb alloy produced by powder metallurgy. Acta Biomater..

[14] Dewidar M. (2010). Microstructure and mechanical properties of biocompatible high density Ti–6Al–4V/W produced by high frequency induction heating sintering. Mater. Des..

[15] Tabrizi S.G., Sajjadi S.A., Babakhani A., Lu W. (2015). Influence of spark plasma sintering and subsequent hot rolling on microstructure and flexural behavior of in-situ TiB and TiC reinforced Ti6Al4V composite. Mater. Sci. Eng. A.

[16] Li X., Zhou Q., Zhao S., Chen J. (2014). Effect of Pulse Current on Bending Behavior of Ti6Al4V Alloy. Procedia Eng..

[17] Banerjee D., Williams J.C. (2013). Perspectives on Titanium Science and Technology. Acta Mater..

[18] Long M., Rack H.J. (1998). Titanium alloys in total joint replacement—a materials science perspective. Biomaterials.

[19] Miura K., Yamada N., Hanada S., Jung T.K., Itoi E. (2011). The bone tissue compatibility of a new Ti–Nb–Sn alloy with a low Young’s modulus. Acta Biomater..

[20] Wang J.L., Liu L.B., Tuo B.Y., Bai W.M., Wang X., Li X., Hu X.P. (2015). Computational Study of Mobilities and Diffusion in Ti-Sn Alloy. J. Phase Equilib. Diffus..

[21] Hsu H.C., Wu S.C., Hong Y.S., Ho W.F. (2009). Mechanical properties and deformation behavior of as-cast Ti–Sn alloys. J. Alloys Compd..

[22] Huan Z.Y.W., Peng G., Wei Z. (2012). Effect of β Stabilizing Elements on the Strengthening Behavior of Titanium α Phase. Rare Met. Mat. Eng..

[23] Zhao Y.Q., Peng G.E. (2014). Current Situation and Development of New Titanium Alloys Invented in China. J. Aero. Mater..

[24] Cai J.M., Cao C.X. (2014). Alloy Design and Application Expectation of A New Generation 600 High Temperature Titanium Alloy. J. Aero. Mater..

[25] Ho W.F., Ju C.P., Lin J.H.C. (1999). Structure and properties of cast binary Ti–Mo alloys. Biomaterials.

[26] Sukedai E., Yoshimitsu D., Matsumoto H., Hashimoto H., Kiritani M. (2003). β to ω phase transformation due to aging in a Ti–Mo alloy deformed in impact compression. Mater. Sci. Eng. A.

[27] Guo H., Enomoto M. (2006). Surface reconstruction associated with α precipitation in a Ti–Mo alloy. Scr. Mater..

[28] Sugano M., Tsuchida Y., Satake T., Ikeda M. (1998). A microstructural study of fatigue fracture in titanium–molybdenum alloys. Mater. Sci. Eng. A.

[29] Zhang L.C., Zhou T., Alpay S.P., Aindow M., Wu M.H. (2005). Origin of pseudoelastic behavior in Ti–Mo-based alloys. Appl. Phys. Lett..

[30] Zhang L.C., Zhou T., Aindow M., Alpay S.P., Blackbur M.J., Wu M.H. (2005). Nucleation of stress-induced martensites in a Ti/Mo-based alloy. J. Mater. Sci..

[31] Liu Y., Wei W.F., Zhou K.C., Chenl F., Tang H.P. (2003). Microstructures and mechanical behavior of PM Ti-Mo alloy. J. Cent. South Univ. Technol..

[32] Oliveira N.T.C., Biaggio S.R., Piazza S., Sunseri C., Di Quarto F. (2004). Photo-electrochemical and impedance investigation of passive layers grown anodically on titanium alloys. Electrochim. Acta.

[33] Zhou Y., Wen S.F., Song B., Zhou X., Teng Q., Wei Q.S., Shi Y.S. (2016). A novel titanium alloy manufactured by selective laser melting: Microstructure, high temperature oxidation resistance. Mater. Des..

[34] Zhang W.F., Huang X., Li X.W., Ma J.M., Cao C.X. (2005). Design Method and Current Research Development of Titanium Alloys. Mater. Rev..

[35] Sun F., Zhang J.Y., Marteleur M., Brozek C., Rauch E.F., Veron M., Vermaut P., Jacques P.J., Prima F. (2015). A new titanium alloy with a combination of high strength, high strain hardening and improved ductility. Scr. Mater..

[36] Abkowitz S., Rowell D. (1986). Superior Fatigue Properties for Blended Elemental P/M Ti-6Al-4V. JOM.

[37] Zhang C.L., Attar H. (2015). Selective Laser Melting of Titanium Alloys and Titanium Matrix Composites for Biomedical Applications: A Review. Adv. Eng. Mater..

[38] Henriques V.A.R., Galvani E.T., Petroni S.L.G., Paula M.S.M., Lemos T.G. (2010). Production of Ti–13Nb–13Zr alloy for surgical implants by powder metallurgy. J. Mater. Sci..

[39] Zheng D., Li X., Li Y., Qu S., Yang C. (2013). ZrO_2_ (3Y) toughened WC composites prepared by spark plasma sintering. J. Alloys Compd..

[40] Borkar T., Nag S., Ren Y., Tiley J., Banerje R. (2014). Reactive spark plasma sintering (SPS) of nitride reinforced titanium alloy composites. J. Alloys Compd..

[41] Yamanoglu R., Gulsoy N., Olevsky E.A., Gulsoy H.O. (2016). Production of porous Ti5Al2.5Fe alloy via pressureless spark plasma sintering. J. Alloys Compd..

[42] Guan A., Sun N. (2017). Synthesis and tribological properties of high purity Ti_2_SC nanolamellas by microwave hybrid heating. J. Alloys Compd..

[43] Tan A., Wang G., Ji L., Tong Y., Duan X.M. (2016). Investigation on 316L/W functionally graded materials fabricated by mechanical alloying and spark plasma sintering. J. Nucl. Mater..

[44] Kim Y., Kim E.-P., Song Y.-B., Lee S.H., Kwon Y.S. (2014). Microstructure and mechanical properties of hot isostatically pressed Ti–6Al–4V alloy. J. Alloys Compd..

[45] Zhang F., Reich M., Kessler O., Burkel E. (2013). The potential of rapid cooling spark plasma sintering for metallic materials. Mater. Today.

[46] Abe J.O., Popoola A.P.I., Popoola O.M. (2020). Consolidation of Ti6Al4V alloy and refractory nitride nanoparticles by spark plasma sintering method: Microstructure, mechanical, corrosion and oxidation characteristics. Mater. Sci. Eng. A.

[47] Abe J.O., Popoola O.M., Popoola A.P.I., Ajenifuja E., Adebiyi D.I. (2019). Application of Taguchi design method for optimization of spark plasma sintering process parameters for Ti-6Al-4V/*h*-BN binary composite. Eng. Res. Express.

[48] Okoroa A.M., Machakab R., Lephuthinga S.S., Okea S.R., Awotundea M.A., Olubambia P.A. (2019). Evaluation of the sinterability, densification behaviour and microhardness of spark plasma sintered multiwall carbon nanotubes reinforced Ti6Al4V nanocomposites. Ceram. Int..

[49] Bolzoni L., Ruiz-Navas E.M., Gordo E. (2013). Influence of sintering parameters on the properties of powder metallurgy Ti–3Al–2.5V alloy. Mater. Charact..

[50] Lu X., Sun B., Zhao T.F., Wang L.N., Liu C.C., Qu X.H. (2014). Microstructure and mechanical properties of spark plasma sintered Ti-Mo alloys for dental applications. Int. J. Miner. Met. Mater..

[51] Lu J.W., Zhao Y.Q., Ge P., Niu H.Z. (2013). Microstructure and beta grain growth behavior of Ti–Mo alloys solution treated. Mater. Charact..

